# Non-mechanical haemodynamic support in acute pulmonary thromboembolism: a scoping review

**DOI:** 10.1186/s40635-025-00793-1

**Published:** 2025-08-18

**Authors:** W. Body, S. Steckle, A. Haggerty, M. Putt, F. Coyer, E. M. Milford

**Affiliations:** 1 Intensive Care, Sunshine Coast University Hospital, Birtinya, QLD Australia; 2 Intensive Care Services, Royal Brisbane and Women’s Hospital, Herston, QLD Australia; 3 The University of Queensland, Brisbane, Australia

**Keywords:** Pulmonary embolism, Haemodynamic support, Preload, Afterload, Inotropy

## Abstract

**Background and aims:**

Acute pulmonary thromboembolism (PE) may require haemodynamic supportive therapies while appropriate therapies for clot burden reduction are pursued. This scoping review aims to identify the non-mechanical haemodynamic support interventions that have been investigated for the management of acute PE, and to map the available evidence for each intervention.

**Methods:**

An iterative search of MEDLINE, Embase, CINAHL and the Cochrane Library was performed to map all available animal studies, case-series, observational studies, human trials, systematic reviews and meta-analyses that investigate any non-mechanical haemodynamic support in acute PE.

**Results:**

6,362 unique articles were screened and of the 132 studies that met the eligibility criteria, 98 were animal studies, 31 human studies, and 3 were systematic reviews. Among all studies 57 different agents were found, including 16 among the human studies. 6 agents were investigated across 7 human randomised controlled trials (RCTs) and included inhaled nitric oxide, fluid, furosemide, diclofenac, sildenafil, and epoprostenol, but were limited to intermediate-risk PE and none demonstrated a mortality benefit from the intervention tested.

**Conclusion:**

The evidence to guide clinical practice in the non-mechanical haemodynamic support of PE is severely limited. However, there are numerous candidate agents that could be further investigated. The high-risk group are an understudied population.

**Supplementary Information:**

The online version contains supplementary material available at 10.1186/s40635-025-00793-1.

## Introduction

Acute pulmonary thromboembolism (PE) occurs when a blood clot becomes lodged in the pulmonary vascular bed obstructing pulmonary blood flow (PBF) [1] by both mechanical obstruction and pulmonary vasoconstriction [2]. Important potential haemodynamic consequences are right ventricular (RV) dysfunction and systemic hypotension [3].

PE can be categorised into either low, intermediate or high risk of mortality. These categories can be identified by either no cardiovascular disturbance (low), or evidence of RV dysfunction either without (intermediate) or with (high) sustained systemic hypotension [3].

While the definitive treatment for acute PE is clot burden reduction, interim haemodynamic support may be required until this can be achieved. Supportive therapies may be mechanical (e.g., extra-corporeal membrane oxygenation) or non-mechanical therapies (e.g., vasoactive medications and fluid therapy) [4, 5]. The latter are the focus of this scoping review.

The European Society of Cardiology (ESC) makes some recommendations on the haemodynamic management of these patients (summarised in appendix 1), but a scoping review to catalogue the full breadth of the evidence base has not previously been performed.

The ideal inotrope, vasopressor and fluid strategy in acute PE is still unknown. The mechanism of many therapies is also unclear and are likely to be multifactorial in many cases. The aim of this scoping review is to identify the interventions that have been investigated in haemodynamic support in acute PE and to map the available evidence for each intervention.

## Methods

JBI methodology for performing and writing a scoping review was followed [6]. The Preferred Reporting Items for Systematic Reviews and Meta-Analyses extension for Scoping Reviews (PRISMA-ScR) was followed where applicable (PRISMA-ScR-Checklist available in supplementary section) [7]. A protocol was developed prior to this review (unpublished) utilising the essential first five stages of the methodological framework for scoping reviews as set out by Arksey and O’Malley [8] with the exception of the optional sixth stage (Consultation Exercise), and are outlined below.

Stage 1—identification of the research question: the 2019 ESC guidelines on haemodynamic management in acute PE, and other clinical resources, revealed a paucity of information on the full scope of potential therapeutic agents and their evidence base (appendix 1). Using the Population, Concept, Context framework for scoping reviews as set out by JBI [6], we developed the following research question: in humans or experimental models of any grade of acute PE (population), what non-mechanical interventions to support haemodynamic status are reported in the literature (concept), in any clinical or pre-clinical setting where the effect on haemodynamic status was evaluated in some way (context). Eligibility criteria were determined prior to implementation of the search except for animal model type, which was refined iteratively during the search to ensure relevance to the research question (Table 1). In answering this question our aims were to produce a catalogue of evidence for non-mechanical haemodynamic support in PE, to provide a list of agents that have been investigated for this purpose, and to provide a brief description of the available evidence for each agent, for the purpose of consolidating and understanding the current scope of literature available, and to aid in future research direction.

**Table 1:** Study eligibility criteria

Inclusion	Exclusion
Population	
• Any acute pulmonary thromboembolism • Any animal model that involves either injected autologous blood clot (formed ex-vivo), or measurable or titratable biologically inert substances injected/inserted to simulate acute pulmonary vascular obstruction (glass beads, microspheres, balloon)* • Any age	• Chronic pulmonary embolism • Acute on chronic pulmonary embolism • Chronic thromboembolic pulmonary embolism (CTEPH) • Primary pulmonary hypertension • Pulmonary arterial hypertension (PAH) • Air embolism • Fat embolism • Amniotic fluid embolism • Other causes of pulmonary hypertension or shock not attributable to acute pulmonary embolism • Animal studies that involve other techniques of simulated PE not outlined in inclusion criteria (in vivo injection of thrombin, collagen and epinephrine cocktail, barium sulfate, muscle cubes, lipiodol), or where acte pulmonary hypertension is induced by other methods (surgical vascular compression/ligation, hypoxia, pharmacological pulmonary vasoconstriction) or models of air, amniotic fluid or fat embolism*
Concept	
• Any pharmacological or fluid therapy given with the intent to improve haemodynamic function	• Mechanical therapies (including ECMO) • Surgical therapies (including embolectomy) • Effect on any measure of haemodynamic function not quantified (or mortality or survival rate not reported)
Context	
• Any pre-clinical or clinical setting • Any quantifiable marker of haemodynamic function including, but not limited to, blood pressure, pulmonary artery pressure, cardiac output, laboratory investigations (including troponin, BNP, lactate), echocardiography, mortality or survival rate is reported	• Effect on any measure of haemodynamic function, mortality or survival rate is not reported
Types of sources of evidence	
• Published meta-analysis, systematic review, randomised-controlled trials (RCTs), cohort studies, case–control studies, case-series • Experimental models, laboratory research and animal studies • Any language • Any publication year (1947 till date of search 30 March 2024)	• Non-peer-reviewed publications • Opinions • Letters • Editorials • Single case-reports

^*^ Eligibility criteria were refined during the screening process, but after implementation of the search strategy

Stage 2—identification of relevant studies: The following databases were searched: Medline (via EBSCO), CINAHL (via EBSCO), Embase, and the Cochrane Library (including CENTRAL and the Cochrane Database of Systematic Reviews), with the most recent search being March 2024. A two-concept search strategy was followed: (A) pulmonary embolism, and (B) haemodynamic support, that were combined using the Boolean operator AND. There were no restrictions set on language or year of publication. The earliest article was from 1947.

An iterative approach to the search strategy was required to ensure inclusion of all interventions potentially interpretable as ‘haemodynamic support’. Initial search terms to conceptualise ‘haemodynamic support’ were identified by reviewing recent publications on this topic (see appendix 1 and 2). Subsequent search terms were identified after reviewing the results of the first search. Citations of relevant recent reviews were also screened to identify other potentially relevant studies. The final search strategies, and iterative search, and their specific dates are presented in appendix 3 to 7 of the supplementary material. Validation of the search strategy was performed to ensure adequate sensitivity for discovering all articles that met the eligibility criteria (appendix 8).

Stage 3—Study selection: Final search results were downloaded to EndNote (v21) and then uploaded to Covidence (www.covidence.org) where the Covidence deduplication was then used. Using the relevant Covidence software tools, two reviewers (WB and SS), blinded to each other, applied the eligibility criteria to first screen all titles and abstracts, and then reviewed all filtered full texts. Non-English articles were translated using Google Translate (https://translate.google.com). Disagreements were reconciled by deliberation between reviewers using the Covidence conflict tool until all conflicts were resolved. Inter-rater reliability was not able to be quantified due to the iterative process of refining eligibility criteria for animal models. There were very few conflicts for selection of human studies. Conflicts for animal study selection were mainly due to unclear eligibility of experimental models until a comprehensive eligibility criteria was agreed upon (Table 1), which could only be finalised once all potential animal models were known, which resolved the remaining conflicts.

Stage 4—Charting the data (data extraction and synthesis): All data extraction was performed by WB. Data extraction was performed manually using Covidence software, then downloaded into Microsoft Excel for further refinement and final sorting. Common to both human and animal studies, extracted data from selected articles included title, authors, year of publication, country of origin, study type, study population, and agents investigated. Additionally, for human studies, a narrative description of study design, PE severity, intervention, comparator, and outcome was collected. Additionally for animal studies, categorical data for species and number, model of PE, whether blinding or randomisation was used, comparator, and the effect direction of each agent (after PE experimental conditions enacted) on cardiac output (CO), pulmonary artery pressure (PAP), pulmonary vascular resistance (PVR) and systemic blood pressure (BP), was collected. For animal experiments, we also allowed the option of collection of pertinent or clarifying findings in a narrative format.

Stage 5—Collating, summarising and reporting the results: Studies were sorted into human and animal studies. We provided a summary overview by reporting a list of all agents studied and corresponding type of evidence (Table 2), as well as a graphical representation of the prevalence of evidence (Table 3). Agents were then categorised by pharmacological action in the main body of the results section. Evidence for each agent was then indexed in the following way:

**Table 2:** Agents investigated for haemodynamic support in acute PE, grouped by available evidence type, listed alphabetically

Study type	Agents investigated
Animal	Adrenomedullin, aminoguanidine, amrinone, l-arginine, aspirin, atropine, BAY41-2272, BAY41-8543, BQ-123, CIBA 31531 Ba, cyproheptadine, diethylenetriamine-nonoate, dobutamine, dopamine, doxycycline, epoprostenol, ETA A-127722, fluid, furosemide, hydralazine, ibuprofen, iloprost, imidazole, indomethacin, inhaled nitric oxide, isoproterenol, ketanserin, ketorolac, levosimendan, meclofenamate, mepyramine, metaraminol, S-methylisothiourea, metiamide, milrinone, N-acetylcysteine, nitroglycerin, nitroprusside, norepinephrine, nitrite, oxygen, PD-145065, phenylephrine, phosphoramidon, PGE2, PGI2, polyphloretin, riociguat, sildenafil, tempol, terlipressin, tezosentan, ZD-2574
Case series	Atropine, dobutamine, dopamine, epinephrine, inhaled nitric oxide, isoproterenol, metaproterenol, norepinephrine
Observational	Dobutamine, epinephrine, fluid, furosemide hydralazine, isoproterenol, ketanserin, levosimendan, PGE1, PGI2, sildenafil
RCT	Diclofenac, epoprostenol, fluid, furosemide, inhaled nitric oxide, sildenafil
Systematic review	Pulmonary vasodilators, inhaled nitric oxide, fluid, furosemide
All studies	Adrenomedullin, aminoguanidine, amrinone, L-arginine, aspirin, atropine, BAY41-2272, BAY41-8543, BM-573, BQ-123, CIBA 31531 Ba, cyproheptadine, diclofenac, diethylenetriamine-nonoate, diltiazem, dobutamine, dopamine, doxycycline, epinephrine, epoprostenol, ETA A-127722, fluid, furosemide, glyceryl-tri-nitrate, hydralazine, ibuprofen, iloprost, imidazole, indomethacin, isoprenaline, ketanserin, ketorolac, levosimendan, meclofenemate, mepyramine, metaproterenol, metaraminol, S-methylisothiourea, metiamide, milrinone, N-acetylcysteine, nitric oxide, nitrite, norepinephrine, PD-145065, phenylephrine, phosphoramidon, polyphloretin, prostaglandin E1, riociguat, sildenafil, sodium nitroprusside, taprostene, tempol, terlipressin, tezosentan, ZD2574

See text for description and source

**Table 3:**
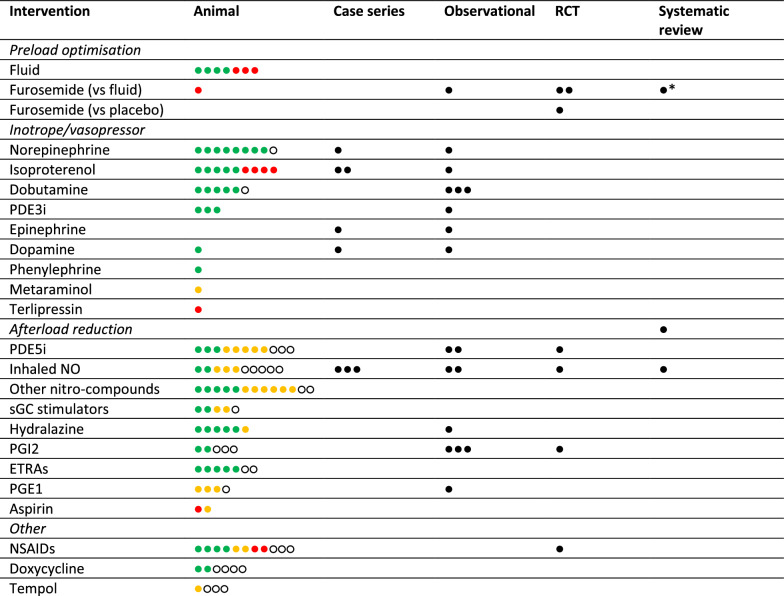
Selected agents investigated for haemodynamic support in acute PE

Each dot ( ) represents an eligible study for each selected agents or groups of agents, where  = increased cardiac output (CO),  = no effect on CO,  = reduced CO, and ○ = unclear effect on CO. ● = prevalence only (one per study)

PDE3i = phosphodiesterase-3 inhibitors: levosimendan, milrinone, amrinone. PDE5i = phosphodiesterase-5 inhibitors: sildenafil. Other nitro-compounds = nitrite, nitroglycerin, nitroprusside. sGC = soluble guanyly cyclase: riociguat, BAY41 compounds. PGE1 = prostaglandin E1. PGI2 = prostaglandin I2: epoprostenol, iloprost, taprostene. NSAIDs = non-steroidal anti-inflammatory drugs: indomethacin, meclofenamate, diclofenac, ketorolac, ibuprofen. ETRAs = endothelin-receptor antagonists: tezosentan, ZD-2574, BQ-123, ETA A-127722. See text for description and source. *Meta-analysis of 3 RCTs and 1 retrospective study [32]

Evidence from animal studies for each agent was grouped by their observed effect on CO, where available. The total number of animal studies investigating and agent was reported, then grouped by the effect on CO, where ‘increased’ and ‘reduced’ were used to mean ‘attenuated’ or ‘exacerbated’ the reduction in CO caused by experimental PE, ‘no effect’ was used to mean a measured but undetected effect on CO, and ‘unclear effect’ was used when CO was either not measured or there was insufficient data available. This was to ensure all discovered articles were indexed in some way here. Other brief commentary was made where pertinent to avoid misrepresentation of overall effect, but in-depth account of each agents’ haemodynamic profile was beyond the scope of this review. Where available, the collected data on CO, PAP, PVR, systemic BP, or other observations, as well as brief details on study design, are presented separately in a table in the supplementary section (appendix 9).

Evidence from human studies for each agent were then indexed in the following order—case-series, observational, RCTs, meta-analysis. PE category (standardised to contemporary categories of ‘intermediate’ or ‘high-risk’ as defined by ESC where possible, noting that most studies were performed prior to the introduction of these categories) and observed outcome from treatment with each agent (if possible to comment) was reported. Emphasis was made on the highest level of human evidence for each agent. Where RCT evidence existed, standard reporting included when and where the study was performed, population, intervention, comparator, and main outcome, unless meta-analysis of these studies was available, in which case a summary of these findings were reported. The absence of any evidence or evidence for ‘high-risk’ PE was highlighted where applicable. A list of all human studies is provided in in the supplementary section (appendix 10).

## Results

### Summary

**Fig. 1:**
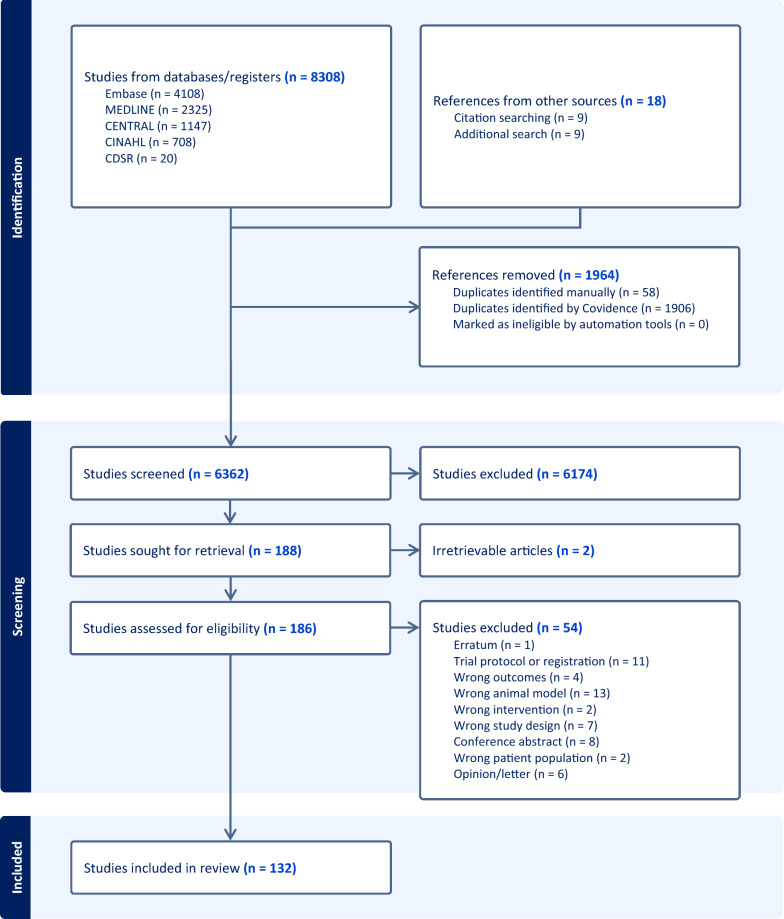
PRISMA flow diagram

The search strategy identified 6362 unique records. After screening and assessing for eligibility, 132 studies were included in the final analysis: 98 animal experiments, 31 human studies (7 RCTs, 17 cohort studies and 7 case series), and 3 systematic reviews (including 1 meta-analysis) (Fig. 1).

Among all human and animal studies, 57 different agents were investigated. In humans, 16 different agents were investigated (Table 2).

There were RCTs for furosemide or fluid [9–11], inhaled nitric oxide [12], intravenous epoprostenol [13], sildenafil [14], and diclofenac [15], all in approximately ‘intermediate-risk’ patients, but none for ‘high-risk’ patients. Although mortality was not the primary outcome in any of these trials, nor were they sufficiently powered to detect a mortality difference, none demonstrated a statistically significant effect on mortality as a secondary outcome. The only agents with trials demonstrating statistically significant benefit in any outcome was furosemide and inhaled nitric oxide. None of these trials observed evidence of harm from the intervention.

Human studies that examined the effect of any agent in patients with ‘high-risk’ PE were limited to case-series [16–22] and cohort studies [23–30].

There were systematic reviews for inhaled nitric oxide [31], pulmonary vasodilators [2] and the use of frusemide or fluid [32].

Table 3 provides a summary of the prevalence of human and animal studies for selected agents by study type and, for animal studies, observed effect on CO, with references for data available in the text below.

### Fluid and diuretics

*Possible mechanism:* Optimise preload by either increasing or reducing RV filling pressure [33].

#### Fluid administration or furosemide

Animal studies: There were seven studies on fluid administration, three showed reduced CO [34–36], and four showed increased CO [37–40]. One of these studies additionally showed that furosemide reduced CO when compared to control animals [40]. Two studies that showed fluid loading increased CO, also showed increase pulmonary oedema [37, 38].

Human studies: One prospective cohort study of 13 patients with acute PE and CI < 2.5 found an increased CI with fluid administration [41]. There were no RCTs examining this. There were four studies investigating furosemide in intermediate-risk PE: (1) a retrospective cohort study of 70 patients [42], (2) two RCTs comparing furosemide to fluid [10, 11], and (3) one RCT comparing frusemide to placebo [9]. A meta-analysis of these trials (total 452 patients) showed that furosemide may improve laboratory [pro-B-type natriuretic peptide (BNP)] and echocardiographic markers of RV dysfunction when compared to either fluid or placebo [32]. An effect of furosemide on mortality compared to either placebo or fluid administration was not demonstrated. There were no trials in high-risk patients.

### Adrenergic agonists

Possible mechanism: Improved cardiac pressure–volume relationship and cardiac output through modulation of heart rate, contractility, pulmonary and systemic vascular resistance, and maintain systemic and coronary perfusion pressure [33].

#### Epinephrine, norepinephrine

Animal studies: Epinephrine: none. Norepinephrine: ten studies, nine showed increased CO [34, 35, 39, 43–48], and one with unclear effect [49].

Human studies: only examined in combination with multiple agents in one retrospective cohort study of 15 patients [26] and one case series of 7 patients [20] with high-risk PE, each with only one death in each cohort.

#### Isoproterenol, dobutamine

Animal studies: Isoproterenol: Nine studies, five showed increased CO [44, 45, 50–52], four showed reduced CO [35, 39, 43, 53]. Dobutamine: Six studies, five showed increased CO [38, 54–57], one with unclear effect [58]. One of these studies showed that despite increased CO, there may be detrimental effect on oxygenation [57].

Human studies: Isoproterenol was reported in two case-series, both published in 1967 where the survival of a total of 12 patients, and no deaths, occurred [16, 21]. There was also a prospective cohort study of nine high-risk PE patients who received isoprenaline, published in 1968, with mixed haemodynamic effect [29].

There were five human studies on dobutamine, including two retrospective cohort studies of six and ten patients with intermediate and high-risk PE that showed increased CI with dobutamine [23, 28], one prospective cohort study of 16 patients with intermediate-risk PE where the four that received dobutamine had increased CI but worse ventilation-perfusion mismatch [59], and two retrospective cohort studies of ‘high-risk’ PE where it was used in combination with multiple other agents [20, 26].

#### Dopamine

Animal studies: One, showed increased CO [54].

Human studies: Only examined in combination with other agents in one retrospective study of 15 patients [26] and one case series of 7 patients [20] with high risk PE, each with only one death in each cohort.

#### Phenylephrine and metaraminol

Animal studies: Phenylephrine: one study showed increased CO [48]. Metaraminol: one study showed no effect on CO [53].

Human studies: nil.

### Vasopressin analogues

Possible mechanism: Increased vascular tone through agonism of argine-vasopressin-1 receptors, increasing systemic and coronary perfusion pressure (provided there is sufficient cardiac output), with relative sparing of the pulmonary vasculature [60, 61].

#### Vasopressin, terlipressin

Animal studies: Vasopressin: none. Terlipressin: one study showed reduced CO but decreased PVR and increased systemic BP [60].

Human studies: nil.

### Phosphodiesterase-3 inhibitors

Possible mechanism: Increase intracellular cyclic guanosine monophosphate (cGMP) and cyclic adenosine monophosphate (cAMP), causing positive inotropy and reduced afterload through pulmonary and systemic vasodilation [62].

#### Amrinone, milrinone, levosimendan

Animal studies: Three studies in total that all showed increased CO, for amrinone [63], levosimendan [64], and one on both milrinone and levosimendan [56].

Human studies: None on milrinone or amrinone. There was one human study on levosimendan, a retrospective cohort study of seven patients with acute PE (six high-risk and one intermediate-risk) who received levosimendan in combination with thrombolysis or heparin [30]. Haemodynamic improvement occurred in all patients following levosimendan, and no patients remained on vasopressors at 12 h. There was one death recorded that occurred on day 3 from PE recurrence.

### Nitro-containing compounds

Possible mechanism: Stimulate guanylate cyclase to increase cGMP, causing pulmonary vasodilation. Inhaled drug delivery has the additional benefit of targeted pulmonary vasodilation, and increased perfusion to the most well-ventilated regions of lung.

#### Inhaled nitric oxide (iNO)

Animal studies: Ten, two showed increased CO [65, 66] and eight showed either no or unclear effect on CO despite all showing reductions in either PVR or PAP [67–74]. 5 of these 10 studies showed either no effect [65, 67, 68, 70, 71] or increase in BP [68]. None showed worsened systemic hypotension by administration of iNO, and three showed reduction in peak troponin concentration [72–74].

Human studies: For high-risk PE, evidence was limited to three case series, all of four patients each, which showed haemodynamic improvement and survival with inhaled nitric oxide [18, 19, 22]. A prospective cohort study included 26 patients with ‘acute right heart syndrome’ of various aetiologies, but only four being due to PE, with only two (of these four) demonstrating an increase in CI with iNO [75].

For intermediate-risk PE, a prospective cohort trial, which served as a pilot study for a subsequent RCT (the iNOPE trial), showed that in eight patients treatment with iNO via nasal prongs was feasible and well tolerated [76].

A 2015 systematic review found only cohort studies, case-series and case-reports, and concluded that of the limited data available, inhaled nitric oxide is probably beneficial in acute PE [31].

A 2019 RCT of 76 patients with intermediate-high risk PE (the iNOPE trial) [12] randomised patients 1:1 to either iNO (via nasal prongs at 50 ppm) or placebo. The intervention was well tolerated but there was no difference found in the primary outcome, a composite of a normal troponin and echocardiogram, but the trial was underpowered. There was, however, an improvement in RV hypokinesis or dilation in a secondary pre-planned post-hoc analysis.

#### Nitrite, nitroprusside, nitroglycerin

Animal studies: Intravenous nitrite: Four studies, all showed increased CO or reduced PVR either alone [77], or in combination with tempol [78] or sildenafil [79], and through various mechanisms [80]. Nitroprusside: eight studies, two showed increased CO [38, 81] and 6 showed either no or unclear effect on CO [82–87]. Of these, three showed reduced systemic BP [82, 83, 87]. Nitroglycerin: one, which showed no effect on CO but reduced PVR [84].

Human studies: Nil.

### Phosphodiesterase-5 inhibitors

Possible mechanism: Similar to PDE3i’s, increase intracellular cGMP causing vasodilation and reduction in RV afterload [14]. Unlike PDE3i’s, there is normally less cardiac effect, but this may be variable depending on the patient population [88].

#### Sildenafil

Animal studies: 11 studies, three showed increased CO [70, 79, 80], and eight showed either no effect [89–93] or unclear effect on CO [94–96]. All 11 studies showed reduced PAP, and relative selectiveness for the pulmonary circulation [89] but some also showed the potential to cause systemic hypotension in this context [93, 96], especially if combined with other vasodilators [79]. N-acetylcysteine was found to potentiate the increased CO effect of sildenafil [92].

Human studies: Two cohort studies showed that sildenafil was well tolerated in intermediate-risk, but not necessarily in high-risk PE [27, 97]. A 2021 small single-centre RCT from Denmark failed to find a haemodynamic benefit [14]. This study included 20 patients with intermediate-high risk PE randomised 1:1 to receive either IV sildenafil 50 mg or placebo. No significant differences were found on assessment using right heart catheterisation, echocardiography, and cardiac MRI.

### Soluble guanylate cyclase (sGC) stimulators

Possible mechanism: Guanylate cyclase is an intermediary in the intracellular mechanism for vasodilation via the NO-sGC-cGMP pathway, that may cause beneficial reduction in RV afterload [2].

#### Riociguat, BAY41-2272, BAY41-8543

Animal studies: Five on this group of drugs, two showed increased CO [98, 99], and three showed either no effect [70, 80] or unclear effect [100] on CO. All showed reduced PVR, and three showed possibility to cause systemic hypotension [80, 99, 100].

Human studies: Nil.

### Hydralazine

Possible mechanism: Pulmonary vasodilation may cause beneficial reduction in RV afterload [2].

#### Hydralazine

Animal studies: Six, five showed increased CO [82–84, 101–103] and one showed no effect on CO [83] and the potential to cause considerable systemic hypotension [83].

Human studies: There was only one human study, a 1983 prospective cohort study that used a pulmonary-artery catheter to measure the response to hydralazine in 26 patients with pulmonary hypertension, 6 of which were due to PE [104]. It showed that systemic hypotension may outweigh any beneficial effect.

### Prostaglandins

Possible mechanism: Pulmonary vasodilation may cause beneficial reduction in RV afterload [2].

#### PGE1 and PGI2 (e.g., epoprostenol, iloprost, taprostene)

Animal studies: PGE1 analogues: All four showed either no effect [83, 84, 105] or unclear effect [106] on CO. Three of these showed reduced systemic BP [83, 105, 106]. Antagonism of PGE1 by polyphloretin was investigated in one that showed no change in CO or systemic BP, but reduced PVR [107]. PGI2 analogues: five studies, two showed increased CO [108, 109] and three showed unclear effect on CO [110–112] One additionally showed PGI2 reduced platelet aggregation and augments lysis by saruplase [112].

Human studies: A 1993 retrospective cohort study used PGE1 in combination with multiple adrenergic agents in 15 patients with high-risk PE, where 14 survived [26]. There were no other human studies.

PGI2 has been more studied in humans. One prospective cohort study of five patients with approximately intermediate-risk PE investigated inhaled PGI2 analogue (iloprost) and demonstrated improved dyspnoea, oxygen saturation, and BNP [113]. Two retrospective cohort studies of inhaled PGI2 (epoprostenol) demonstrated feasibility of this intervention in acute PE for RV dysfunction [25, 114], but the larger of these trials, where 48 of the 111 included patients had high-risk PE, found 14% experienced decreased systemic BP requiring additional vasoactive therapy [25].

A 2010 Dutch single-centre RCT investigation of PGI2 in 14 patients with intermediate-risk PE [13] randomised patients 1:1 to receive either intravenous epoprostenol 1–4 ng/kg/min or placebo. There were no differences in any haemodynamic parameter assessed by echocardiography and laboratory measurements.

### Endothelin receptor antagonists

Possible mechanism: Antagonism of the vasoconstrictor endothelin released from the pulmonary vascular endothelium in acute PE, attenuating pulmonary vasoconstriction [115]. Additionally, may counteract coronary vasoconstriction [116].

#### Tezosentan, ZD-2574, BQ-123, ETA A-127722, phosphoramidon

Animal studies: Seven studies, three showed increased CO [65, 117, 118], and four showed either no effect [115, 119] or unclear effect [116, 120] on CO. One of these showed attenuation of reduced coronary blood flow [116].

Human studies: nil.

### Serotonin, histamine, and thromboxane antagonists

Possible mechanism: Antagonism of vasoconstrictive mediators released by activated platelets in acute PE, attenuating pulmonary vasoconstriction [2].

#### Imidazole, ketanserin, chlorpheniramine, metiamide, cyproheptadine, BM-573, Mepyramine

Animal studies: Imidazole: one, showed increased CO, reduced PVR and no change in systemic BP [108]. Ketanserin, chlorpheniramine, metiamide: one study each, showed no effect on CO or systemic BP but reduced PVR and PAP [107, 121]. Cyproheptadine: one, unclear effect on CO but showed reduced PAP and increased systemic BP [122]. BM-573: one, showed unclear effect on CO or systemic BP, but reduced PVR and PAP [123]. Mepyramine: one, showed unclear effect on CO or systemic BP but reduced PAP [110].

Human studies: One prospective cohort study of ten patients with intermediate and high-risk PE were given ketanserin. CO was unchanged, but both PAP and systemic BP were reduced [24].

### Non-steroidal anti-inflammatory drugs (NSAIDs)

Possible mechanism: Reduction in inflammatory mediators such as cyclo-oxygenase and cytokine-induced neutrophil chemoattractant (CINC), and reduced neutrophil influx, may reduce RV dysfunction in acute PE [15, 124].

#### Indomethacin, ketorolac, ibuprofen, meclofenamate, diclofenac

Animal studies*:* Haemodynamic effect by this group of drugs is mixed. Indomethacin: four, one showed increase CO [108], one showed decreased CO [125], and two showed unclear effect on CO with opposite effects on PAP [110, 126]. Ketorolac: two, one showed increased CO [124], and one showed unclear effect on CO but increase systemic BP [127]. Ibuprofen: one, which showed increased CO, reduced PVR and no change in systemic BP [128]. Meclofenemate: two, one showed increased CO [128] and the other showed no change in CO [107], and both showed reduced PVR and no change in systemic BP.

Human studies: Diclofenac was investigated in a single-centre RCT in Spain in 2018 [15]. Thirty-four patients with intermediate-risk PE were randomised 1:1 to receive either intravenous diclofenac 75 mg or placebo. There were no differences in echocardiographic or other data, but the trial was underpowered due to lower recruitment than anticipated.

#### Aspirin

Animal studies: Two, one showed reduced CO and increased PVR [125], and one showed unclear effect on CO but increased PAP [69].

Human studies: nil.

### Matrix metallo-proteinase (MMP) inhibitors and antioxidants

Possible mechanism: Doxycycline as an MMP antagonist and tempol as an antioxidant may counteract the cell-damaging effects of upregulated MMP and reactive oxygen species (ROS) that contribute to RV dysfunction during acute PE [129, 130].

#### Doxycycline and tempol

Animal studies: Doxycycline: six, three studies showed increased CO or survival [55, 129, 131], and three showed unclear effect on CO [132–134]. Of these, four showed reduced PVR [55, 129, 133], and one showed increased systemic BP [132]. Tempol: four, none demonstrated effect on CO but all showed reduce PAP [78, 85, 130, 135].

Human studies: nil.

### Other

Animal studies investigating other agents which found no effect on CO include aminoguanidine [136], *N*-acetylcysteine (unless combined with sildenafil) [92], and methylisothiourea [91]. Other agents with unclear effect on CO include l-arginine [94, 137–139], CIBA 31531 Ba [140], atropine [49], and diethylenetriamine nonoate [95]. There was one study on adrenomedullin which showed increased CO but decreased systemic BP [141]. There was one study on diltiazem that showed unclear effect on CO but increased systemic BP [86].

## Discussion

We performed a broad search for any potential therapy investigated as a non-mechanical haemodynamic support in acute PE and catalogued the available evidence for each agent identified.

The identified human clinical trials were mostly small and limited to patient populations with intermediate risk PE. While these provide some evidence on the effects of various interventions on physiological endpoints, their results cannot necessarily be generalised to patient centred outcomes in high-risk PE. In addition, finding a difference in an outcome measure such as mortality in the intermediate-risk group would likely require unfeasibly large sample sizes due to the lower baseline mortality in this group compared to the high risk group. It is, however, the high risk group that are most likely to benefit from appropriate intervention.

The majority of studies identified by this review were hypothesis generating animal and observational studies, with numerous agents that have never been tested in this context in human subjects.

Preload optimisation—While there was no difference in mortality in human RCTs (which may be due to a type 2 error), furosemide may have a small benefit on surrogate markers of haemodynamic status when compared to fluid administration, but these findings were inconsistent between studies [32]. However, it unclear whether the effects demonstrated in these studies are due to furosemide or simply less fluid administration, and why there is inconsistency with observed effects in animal studies.

Inotropes/vasopressors—Despite recommendation for the use of norepinephrine in the ESC guidelines [3] (appendix 1), evidence for its use is mainly from animal studies [34, 35, 39, 43–48]. Observational human data where it is used in combination with multiple other agents makes interpretation of its specific effect impossible. Human data for other adrenergic agonists were similarly limited.

Vasopressin analogues have the theoretical advantage of causing vasoconstriction of the systemic circulation in excess of the pulmonary circulation, resulting in potential augmentation of myocardial perfusion without a detrimental increase in RV afterload [60]. Although the single animal study on this group of agents demonstrated a decrease in PVR and increase in systemic vascular resistance, the overall effect was a decrease in CO and worsening of systemic perfusion [60]. However, there remains unanswered questions around optimal dosing or combinations with other therapies.

RV afterload reduction—Most therapies identified in our review targeted RV afterload reduction. Despite frequently demonstrated beneficial effects in animal models (Table 3), this has not yet translated to a clear demonstration of benefit in human trials [12–14]. This may be due to the greater heterogeneity and complexity in haemodynamic status and pre-existing disease in the clinical population, as well as the small sample sizes of these trials due to the relative rarity of the disease resulting in the possibility of a type 2 error. However, numerous examples demonstrate that any potential benefit from RV afterload reduction with these agents may be offset by the potential for worsening of shock from systemic vasodilation [24, 25, 104], and warrants careful further evaluation.

PDE3i’s have the theoretical advantage of both positive inotropy and RV afterload reduction. We found three animal studies, all showing beneficial haemodynamic effects [56, 63, 64], and only one human study, a retrospective cohort study of only 7 patients that used levosimendan that also suggested benefit [30]. It is, however, unclear if the dual mechanism of action of these agents is superior to separately titratable agents that allow more precise targeting of individual goals.

Other—Agents that may reduce myocardial cellular dysfunction include NSAIDs through their anti-inflammatory action. The data from animal studies are mixed [107, 108, 124, 126–128], and the only clinical trial was ultimately underpowered with an equivocal result [15]. MMP inhibitors and anti-oxidants are similarly under-studied, with only animal data available but all of these studies showed beneficial effects on haemodynamic function [55, 78, 85, 129–135].

### Limitations

Limitations common to scoping reviews that are applicable to this report include omissions, misrepresentations of study validity, and limited appraisal, due to the aim of reporting breadth rather than depth. As such, scoping reviews are often hypothesis generating rather than hypothesis testing. However, an implicit aim of this review is to provide direction for more focussed systematic reviews and primary research.

Potential sources of article omissions from our search include the limitations that were required for the search strategy to maintain feasibility and the possibility of unidentified haemodynamic support agents not included in the searches despite the iterative method. Prior to implementation, we tested our search strategy to ensure adequate sensitivity (appendix 8). Following our search, potential omissions, as well as selection bias, was reduced by having two reviewers independently apply eligibility criteria, and requirement for bilateral agreement by reviewers for exclusion of articles.

A recognised omission was oxygen as a haemodynamic intervention. Studies investigating oxygen therapy as a haemodynamic support for pulmonary vasodilation in PE were noted [142] during the iterative process of defining ‘haemodynamic support’, but ultimately this intervention was not included due to incorporation into our search strategy becoming unfeasible. Given oxygen’s ubiquity, it was felt a different search strategy would be required to investigate its use specifically in haemodynamic support.

Eligible animal models for inclusion was limited to those that employed embolization of ex-vivo formed autologous blood clot or controlled embolization of a biologically inert material as this was felt to be the most clinically relevant. These were also the most commonly used models, and criticisms of other animal models are their high mortality rate, uncontrollable size of clot, or being too unlike human pathophysiology [143]. In total, after screening, 13 articles were excluded due to ‘wrong animal model’ (i.e., fit one of the descriptions listed in the exclusion criteria, which was determined by reviewers after implementation of the search strategy (during full text review and before data extraction). However, besides expert commentary that guided our exclusion criteria [143], we identified a gap in the literature on review and appraisal of experimental models of acute PE, which could form the basis of a separate study.

Care should be taken in interpretation of potential clinical utility of reported agents. Due to the required brevity of describing individual studies to deliver the aimed breadth, appraisal of these studies is beyond the scope of this review. Additionally, care should be taken in interpreting reported increases in CO as purely beneficial, for example if it is accompanied by pulmonary oedema as may be the case with fluid administration [37], systemic hypotension as may be the case with hydralazine [104], or worsened pulmonary shunt fraction as may be the case with dobutamine [57].

### Future directions

There are many candidate therapies for further investigation. Vasopressin, noradrenaline, dobutamine, milrinone, levosimendan, nitric oxide, PGI2, or sildenafil would appear to have the greatest clinical applicability based on an individual clinician’s assessment of utility in a given case, as these agents are already frequently used in critical care environments. Fluid administration and its potential harms needs further clarification in carefully designed experiments. When designing animal experiments, it would be useful to include controls for both the effects of anaesthetic, and haemodynamic evolution of experimental PE left untreated, such as demonstrated by Dias-Junior and colleagues [77]. Since vasodilators are a promising potential supportive therapy, key to assessing potential clinical utility is evaluating its selectivity for the pulmonary circulation, for example reporting on PVR/SVR ratio as numerous studies have done [71, 77, 84].

Antioxidants, NSAIDs, and MMP inhibitors are intriguing areas of research and their utility in human PE has been little tested. These drugs are commonly used by clinicians for other purposes. Their use in haemodynamic correction is interesting because it focuses on underlying biological mechanisms for failure rather than more direct attempts at manipulation of haemodynamic components.

Studying the high-risk population in human trials is challenging due to the difficulties around consenting and enrolling critically unwell patients into clinical trials and competing clinical priorities. As noted above, the population studied in clinical trials of acute PE so far are the intermediate-risk group, possibly, at least in part, due to these barriers. Laboratory markers and echocardiographic data would be useful surrogate measurements evaluating haemodynamic response to intervention since, by definition, these patients are not hypotensive, and the risks of pulmonary artery catheter insertion may not be clinically justified. The high-risk group, however, may be the most likely to benefit from any interventions that improves haemodynamic function as a bridge to clot resolution. Hence, efforts to include this group in future trials are warranted.

## Conclusion

Acute PE is a life-threatening condition that despite recent advances still has a high mortality rate due to respiratory and haemodynamic compromise. This scoping review identified 57 different agents that have been investigated for the non-mechanical haemodynamic support of acute PE as a bridge to definitive clot resolution. Nearly all studies identified were of an insufficient grade to reliably inform clinical practice, but there were several agents that showed potential beneficial effects that could be further investigated in sufficiently powered clinical trials. We also found that the high-risk group were an understudied population despite being the most likely to benefit from supportive care as a bridge to definitive treatment.

## Take home message

57 drugs have been investigated for haemodynamic support in acute pulmonary thromboembolism, including 6 in human randomised controlled trials. There are multiple potential therapeutic targets, but none have been subject to clinical trials in the sickest patients with high-risk PE, therefore, their utility remains uncertain.

## Supplementary Information

Supplementary Material 1.

Supplementary Material 2.

Supplementary Material 3.

## Data Availability

The data supporting the findings of this review are available from the original published articles indicated in the reference list.
