# Supplementary material for: Non-mechanical haemodynamic support in acute pulmonary thromboembolism: a scoping review
Source: Intensive Care Med Exp. 2025 Aug 18;13:85. doi: 10.1186/s40635-025-00793-1 (PMC12361032; doi:10.1186/s40635-025-00793-1)
Supplement: Supplementary file 1 — Supplementary Material 1. [file 40635_2025_793_MOESM1_ESM.docx]

## Supplementary material

### Appendix 1. Summary of guidelines and POC resources

Table of comments and language used regarding haemodynamic support by major guidelines and point-of-care (POC) resources, including indication of strength of recommendation, reference, or authority.

| **Guideline** | **Comments and language used regarding haemodynamic support** | **Strength of guidelines/qualifying comments** |
| --- | --- | --- |
| ESC 2019[[3](#_ENREF_3)] | Chapter 6.1.2  “**Volume** optimisation:  “Cautious **volume** loading, saline or Ringer’s lactate, </=500mL over 15-30min… consider in… normal-low CVP (… concomitant hypovolaemia)”  “..can over-distend… RV, worsen ventricular interdependence, and reduce CO”  “**Vasopressors** and **inotropes**:  “**Norepinephrine** 0.2-1.0mcg/kg/min” “Increase RV inotropy and SBP, promote positive ventricular interaction… restore coronary perfusion gradient”  “Excessive vasoconstriction may worsen tissue perfusion”  “**Dobutamine**, 2-20mcg/kg/min “Increase RV inotropy, lower filling pressures… may aggravate arterial hypotension… or arrhythmias”  “Use of **vasopressors** is often necessary”  “**Norepinephrine** can improve systemic haemodynamics”  “**Dobutamine** may be considered”  “**Levosimendan** may restore RV-pulmonary arterial coupling… no evidence of clinical benefit is available”  “**Vasodilators** decrease PAP and PVR, but may worsen hypotension”  “Small clinical studies have suggested **inhaled nitric oxide** may improve haemodynamic status… no evidence of clinical efficacy is available to date” | Use of Norepinephrine and dobutamine Grade IIa, level C  No grade of recommendation for fluid, levosimendan, vasodilators, or iNO.  Comments on volume loading reference non-systematic review[[41](#_ENREF_41)].  Comments on norepinephrine reference animal study[[34](#_ENREF_34)].  Comments on dobutamine reference cohort study[[59](#_ENREF_59)].  Comments on levosimendan reference animal study[[64](#_ENREF_64)].  Comments on iNO reference a systematic review[[31](#_ENREF_31)]. |
| NICE 2020[[145](#_ENREF_145)] | None. | n/a |
| CHEST 2021[[146](#_ENREF_146)] | None. | n/a |
| ASH 2020[[147](#_ENREF_147)] | None. | n/a |
| **Point-Of-Care Resources** |  |  |
| BMJ Best Practice.  Updated 08/2022.  Accessed 22/01/24. | “Seek senior help and escalate to critical care”.  “Give IV **fluids** if SBP < 90mmHg and the JVP is not elevated.”  “Either saline or Hartmann’s. A cautious **fluid** challenge over 15-30mins.”  “Monitor for signs of heart failure. The leading cause of death in high-risk PE is acute RV failure.” | References ESC Guidelines 2019 |
| UpToDate | Provides summary of ESC recommendations, without any notable differences. | References ESC Guidelines 2019. |
| MedScape  Updated 09/2020.  Accessed 22/01/2024 | “IV **fluids** may help or may hurt”  “A cautious trial of a small **fluid** bolus may be attempted” | No reference provided. |
| DynaMed  Updated 12/2023  Accessed 22/01/2024 | “Cautious **volume** loading… </=500mL over 15-30mins [if suspected] concomitant hypovolaemia… aggressive **volume** loading can reduce CO”.  “**Vasopressors** often needed”  “**Norepinephrine** 0.2-1mcg/kg/min may be considered”  “**Dobutamine** 2-20mcg/kg/min may be considered”. | References ESC Guidelines 2019. |

### Appendix 2. Key terms and their source identified prior to initial search strategy

| **Source** | **Terms used in descriptions of haemodynamic supports** |
| --- | --- |
| ESC Guidelines 2019 [[3](#_ENREF_3)] | Volume loading, saline, ringer’s lactate, vasopressors, inotropes, fluid challenge, norepinephrine, dobutamine, levosimendan, vasodilators, nitric oxide |
| Piazza JACC 2020 [[4](#_ENREF_4)] | Intravenous volume, preload, vasopressors, inotropes, haemodynamic support, norepinephrine, epinephrine, dopamine, dobutamine, vasopressin, phenylephrine, pulmonary vasodilators, inhaled nitric oxide |
| Lyhne et al. Pulm Circ. 2020 [[2](#_ENREF_2)] | vasodilator, hydralazine, phosphodiesterase inhibitors, sildenafil, soluble guanylyl cyclase, riociguat, endothelin receptor antagonist, nitric oxide, nitroprusside, nitroglycerin, prostaglandins, epoprostenol, antihistamine, NSAID, diclofenac, cinaciguat |
| Sifuentes et al. Int Card Clin. 2023 [[5](#_ENREF_5)] | Fluid resuscitation, volume loading, decongestion, furosemide, diuretics, vasopressors, inotrope, epinephrine, norepinephrine, vasopressin, phenylephrine, dobutamine, milrinone, pulmonary vasodilators, nitric oxide, epoprostenol |
| Perez-Nieto et al. Frontiers 2023 [[148](#_ENREF_148)] | Intravenous fluids, volume expansion, fluid, diuretics, furosemide, sodium chloride, vasopressor, norepinephrine, vasoconstrictor, dobutamine, levosimendan, nitric oxide, soluble guanylate cyclase, cyclic guanylate monophosphate, prostanoid, endothelin, sildenafil, phosphodiesterase 5 inhibitor, epoprostenol, prostacyclin, vasodilator, hydralazine |
| (Various) | Meclofenemate, polyphloretin, clopheniramine and metiamide [[107](#_ENREF_107)]  Cinaciguat, BAY 41-8543 [[99](#_ENREF_99)]  BAY 41-2272 [[100](#_ENREF_100)]  ABT-627 [[149](#_ENREF_149)]  Ibuprofen [[128](#_ENREF_128)]  nstpbp5185 [[150](#_ENREF_150)]  terlipressin [[60](#_ENREF_60)] |
| Other agents selected by authors not included elsewhere | Ambrisentan, dipyridamole, enoximone, amrinone |

### Appendix 3. Search strategy: MEDLINE via EBSCO

Hits: 2325

Date performed: 28 March 2024

| Search line | Search | Results (n) |
| --- | --- | --- |
| S1 | MH pulmonary embolism+ OR TI (“pulmonary” OR “lung” OR “venous*”) N2 (“embol*” OR “thromb*” OR “clot”) OR TI “pulmonary-embol*” OR TI “pulmonary-thromb*” OR TI “lung-thromb*” OR TI “lung-embol*” OR TI “venous-embol*” OR TI “venous-thromb*” OR TI “venothromb*” OR TI “venoembol*” OR TI “VTE” OR TI “PE” | 78,957 |
| S2 | MH diuretics+ OR MH furosemide OR AB “diure*” OR AB “furosemide” OR AB “frusemide” OR TI “fluid*” OR TI “volume” | 321,481 |
| S3 | MH vasodilator agents+ OR MH hydralazine OR AB vasodilat* OR AB hydralazine* OR AB hydrallazin* OR AB hydrazinophtalazine OR AB hydrazinophthalazine OR AB hydrazinophtalizine OR AB dralzine OR AB hydralacin OR AB hydralazine OR AB “hypophthalin” OR AB “hypoftalin” OR AB “hydrazinophthalazine” OR AB “1‐hydrazinophthalazine” OR AB “apressin” OR AB “nepresol” OR AB “apressoline” OR AB “apresoline” OR AB “apresolin” OR AB “alphapress” OR AB “alazine” OR AB “idralazina” OR AB “lopress” OR AB “plethorit” OR AB “praeparat” | 105,476 |
| S4 | MH nitric oxide OR MH nitroprusside OR MH nitroglycerin OR AB “nitric*” OR AB “inhaled-nitr*” OR AB “nitroprussid*” OR AB “nitro-prussid*” OR AB “sodium-nitr*” OR AB “nitrat*” OR AB “glyceryltrin*” OR AB “glyceryl-trin*” OR AB “tri-nitr*” OR AB “glyceryl-tri-nitr*” OR AB “nitroglycer*” OR AB “nitro-glycer*” | 281,276 |
| S5 | MH prostaglandins+ OR MH epoprostenol OR MH iloprost OR AB “prostaglandin*” OR AB “epoprostenol” OR AB “prostacyclin*” OR AB “prosta-cyclin*” OR AB “prostanoid*” OR AB “prosta-noid*” OR AB “iloprost” OR AB “treprostinil” OR AB “selexipag” | 147,427 |
| S6 | MH phosphodiesterase inhibitors+ OR MH sildenafil citrate OR MH tadalafil OR MH dipyridamole OR AB “phosphodiesterase*” OR AB “phospho-diest*” OR AB “phospho-di-est*” OR AB “sildenafil” OR AB “tadalafil” OR AB “dipyridamole” OR AB “persantin*” | 54,145 |
| S7 | MH endothelin receptor antagonists+ OR MH bosentan OR AB “endothelin receptor*” OR AB “bosentan” OR AB “macitentan” OR AB “ambrisentan” | 9,175 |
| S8 | MH soluble guanylyl cyclase+ OR AB “guanylyl*” OR AB “guanylat*” OR AB “riociguat*” | 16,207 |
| S9 | MH histamine antagonists+ OR MH chlorpheniramine OR AB “histamin*” OR AB “antihistamin*” OR AB “anti-histamin*” OR AB “chlorphen*” | 81,610 |
| S10 | MH anti-inflammatory agents, non-steroidal+ OR MH diclofenac OR MH ibuprofen OR MH meclofenamic acid OR AB “meclofen*” OR AB “diclofena*” OR AB “ibuprofen*” OR AB “NSAID*” OR AB “non-steroid* anti*” OR AB “nonsteroid* anti*” | 134,786 |
| S11 | MH Vasoconstrictor agents OR MH vasopressins OR MH terlipressin OR MH phenylephrine OR AB “vasopressor*” OR AB “Vaso-pressor*” OR AB “vasopressin*” OR AB “arginine-vasopress*” OR AB “terlipressin*” OR AB “phenylephrin*” | 91,064 |
| S12 | MH cardiotonic agents+ OR MH catecholamines+ OR MH adrenergic agonists+ OR MH dopamine OR MH epinephrine OR MH norepinephrine OR MH dobutamine OR MH milrinone OR MH simendan OR AB “catehcolamin*” OR AB “inotrop*” OR AB “adrenalin*” OR AB “epinephrin*” OR AB “noradrenalin*” OR AB “nor-adrenalin*” OR AB “norepinephrin*” OR AB “nor-epinephrin*” OR AB “dopamin*” OR AB “dobutamin*” OR AB “milrinon*” OR AB “simendan*” OR AB “levosimendan*” | 460,605 |
| S13 | S2 OR S3 OR S4 OR S5 OR S6 OR S7 OR S8 OR S9 OR S10 OR S11 OR S12 | 1,489,763 |
| S14 | S1 AND S13 | 2,325 |

### Appendix 4. Search strategy: CINAHL via EBSCO

Hits: 708
Date performed: 30 March 2024

| Search line | Search | Results (n) |
| --- | --- | --- |
| S1 | MM pulmonary embolism OR TI (“pulmonary” OR “lung” OR “venous*”) N2 (“embol*” OR “thromb*” OR “clot”) OR TI “pulmonary-embol*” OR TI “pulmonary-thromb*” OR TI “lung-thromb*” OR TI “lung-embol*” OR TI “venous-embol*” OR TI “venous-thromb*” OR TI “venothromb*” OR TI “venoembol*” OR TI “VTE” OR TI “PE” | 20,468 |
| S2 | MH diuretics+ OR MH furosemide OR AB “diure*” OR AB “furosemide” OR AB “frusemide” OR TI “fluid*” OR TI “volume” | 61,708 |
| S3 | MH vasodilator agents+ OR MH hydralazine OR AB vasodilat* OR AB hydralazine* OR AB hydrallazin* OR AB hydrazinophtalazine OR AB hydrazinophthalazine OR AB hydrazinophtalizine OR AB dralzine OR AB hydralacin OR AB hydralazine OR AB “hypophthalin” OR AB “hypoftalin” OR AB “hydrazinophthalazine” OR AB “1‐hydrazinophthalazine” OR AB “apressin” OR AB “nepresol” OR AB “apressoline” OR AB “apresoline” OR AB “apresolin” OR AB “alphapress” OR AB “alazine” OR AB “idralazina” OR AB “lopress” OR AB “plethorit” OR AB “praeparat” | 28,014 |
| S4 | MM nitric oxide OR MH nitroprusside OR MH nitroglycerin OR AB “nitric*” OR AB “inhaled-nitr*” OR AB “nitroprussid*” OR AB “nitro-prussid*” OR AB “sodium-nitr*” OR AB “nitrat*” OR AB “glyceryltrin*” OR AB “glyceryl-trin*” OR AB “tri-nitr*” OR AB “glyceryl-tri-nitr*” OR AB “nitroglycer*” OR AB “nitro-glycer*” | 22,086 |
| S5 | MH prostaglandins+ OR MM epoprostenol OR MM iloprost OR AB “prostaglandin*” OR AB “epoprostenol” OR AB “prostacyclin*” OR AB “prosta-cyclin*” OR AB “prostanoid*” OR AB “prosta-noid*” OR AB “iloprost” OR AB “treprostinil” OR AB “selexipag” | 11,803 |
| S6 | MH phosphodiesterase inhibitors+ OR MM sildenafil OR MM tadalafil OR MM dipyridamole OR AB “phosphodiesterase*” OR AB “phospho-diest*” OR AB “phospho-di-est*” OR AB “sildenafil” OR AB “tadalafil” OR AB “dipyridamole” OR AB “persantin*” | 6,155 |
| S7 | MH endothelin receptor antagonists+ OR MM bosentan OR AB “endothelin receptor*” OR AB “bosentan” OR AB “macitentan” OR AB “ambrisentan” | 853 |
| S8 | AB “guanylyl*” OR AB “guanylat*” OR AB “riociguat*” | 623 |
| S9 | MH histamine antagonists+ OR MM chlorpheniramine OR AB “histamin*” OR AB “antihistamin*” OR AB “anti-histamin*” OR AB “chlorphen*” | 10,012 |
| S10 | MH antiinflammatory agents, non-steroidal+ OR MH diclofenac OR MH ibuprofen OR AB “meclofen*” OR AB “diclofena*” OR AB “ibuprofen*” OR AB “NSAID*” OR AB “non-steroid* anti*” OR AB “nonsteroid* anti*” | 41,091 |
| S11 | MH Vasoconstrictor agents OR MH vasopressins OR MM phenylephrine OR AB “vasopressor*” OR AB “Vaso-pressor*” OR AB “vasopressin*” OR AB “arginine-vasopress*” OR AB “terlipressin*” OR AB “phenylephrin*” | 10,121 |
| S12 | MH cardiotonic agents+ OR MH catecholamines+ OR MH adrenergic agents+ OR MM epinephrine OR MM norepinephrine OR MM dobutamine OR MM dopamine OR MM milrinone OR AB “catehcolamin*” OR AB “inotrop*” OR AB “adrenalin*” OR AB “epinephrin*” OR AB “noradrenalin*” OR AB “nor-adrenalin*” OR AB “norepinephrin*” OR AB “nor-epinephrin*” OR AB “dopamin*” OR AB “dobutamin*” OR AB “milrinon*” OR AB “simendan*” OR AB “levosimendan*” | 72,562 |
| S13 | S2 OR S3 OR S4 OR S5 OR S6 OR S7 OR S8 OR S9 OR S10 OR S11 OR S12 | 229,099 |
| S14 | S1 AND S13 | 708 |

### Appendix 5. Search strategy: Embase

Hits: 4108 (Medline = 2,325, Non-Medline = 1,782, Pre-print = 1)
Date performed: 28 March 2024

| Search line | Search | Results (n) |
| --- | --- | --- |
| #1 | ((('lung' OR 'pulmonary' OR 'venous') NEAR/2 ('embol*' OR 'thromb*' OR 'clot')):ti) OR 'pulmonary-embol*':ti OR 'pulmonary-thromb*':ti OR 'lung-thromb*':ti OR 'lung-embol*':ti OR 'venous-embol*':ti OR 'venous-thromb*':ti OR 'venothromb*':ti OR 'venoembol*':ti OR 'veno-thromb*':ti OR 'veno-embol*':ti OR 'pe':ti OR 'vte':ti | 85,485 |
| #2 | 'lung embolism'/exp/mj | 43,824 |
| #3 | 'diuretic agent'/exp/mj | 163,445 |
| #4 | 'diure*':ti,ab OR 'frusemide':ti,ab OR 'furosemide':ti,ab OR fluid:ti OR volume:ti | 356,437 |
| #5 | 'hydralazine'/exp/mj | 8,527 |
| #6 | 'vasodilat*':ti,ab OR hydralazin*:ti,ab OR hydrallazin*:ti,ab OR hydrazinophtalazine:ti,ab OR hydrazinophtalizine:ti,ab OR dralzine:ti,ab OR hydralacin:ti,ab OR hydralazine:ti,ab OR hypophthalin:ti,ab OR hypoftalin:ti,ab OR hydrazinophthalazine:ti,ab OR 1‐hydrazinophthalazine:ti,ab OR apressin:ti,ab OR nepresol:ti,ab OR apressoline:ti,ab OR apresoline:ti,ab OR apresolin:ti,ab OR alphapress:ti,ab OR alazine:ti,ab OR idralazina:ti,ab OR lopress:ti,ab OR plethorit:ti,ab OR praeparat:ti,ab | 102,964 |
| #7 | 'nitric oxide'/exp/mj OR 'nitroprusside sodium'/exp/mj OR 'glyceryl trinitrate'/exp/mj | 86,630 |
| #8 | nitric*:ti,ab OR nitroprussid*:ti,ab OR 'prussid*':ti,ab OR nitrat*:ti,ab OR nitroglycer*:ti,ab OR 'nitro-glycer*':ti,ab OR 'glyceryl*':ti,ab | 331,084 |
| #9 | 'prostanoid'/exp/mj OR 'iloprost'/exp/mj OR 'prostacyclin'/exp/mj | 101,698 |
| #10 | prostaglandin*:ti,ab OR epoprostenol:ti,ab OR prostacyclin*:ti,ab OR prostanoid*:ti,ab OR iloprost:ti,ab OR treprostinil:ti,ab OR selexipag:ti,ab | 147,347 |
| #11 | 'phosphodiesterase inhibitor'/exp/mj | 93,010 |
| #12 | 'phosphodiesterase inhibitor'/exp/mj OR phosphodiesterase*:ti,ab OR 'phospho diest*':ti,ab OR 'phospho di est*':ti,ab OR sildenafil:ti,ab OR tadalafil:ti,ab OR dipyridamole:ti,ab OR persantin*:ti,ab | 64,044 |
| #13 | 'endothelin receptor antagonist'/exp/mj | 5,730 |
| #14 | 'endothelin receptor*':ti,ab OR bosentan:ti,ab OR macitentan:ti,ab OR ambrisentan:ti,ab | 10,699 |
| #15 | 'guanylate cyclase'/exp/mj | 4,966 |
| #16 | guanylyl*:ti,ab OR guanylat*:ti,ab OR riociguat*:ti,ab | 21,392 |
| #17 | 'antihistaminic agent'/exp/mj | 134,326 |
| #18 | histamin*:ti,ab OR antihistamin*:ti,ab OR 'anti histamin*':ti,ab OR chlorphen*:ti,ab | 102,172 |
| #19 | 'nonsteroid antiinflammatory agent'/exp/mj | 501,264 |
| #20 | meclofen*:ti,ab OR diclofena*:ti,ab OR ibuprofen*:ti,ab OR nsaid*:ti,ab OR 'non-steroid* anti*':ti,ab OR 'nonsteroid* anti*':ti,ab | 116,719 |
| #21 | 'vasopressin'/exp/mj OR 'terlipressin'/exp/mj OR 'phenylephrine'/exp/mj | 32,315 |
| #22 | vasopressin*:ti,ab OR 'arginine vasopress*':ti,ab OR terlipressin*:ti,ab OR phenylephrin*:ti,ab | 68,752 |
| #23 | 'cardiotonic agent'/exp/mj OR 'catecholamine'/exp/mj OR 'adrenergic receptor stimulating agent'/exp/mj OR 'dopamine receptor stimulating agent'/exp/mj OR 'milrinone'/exp/mj OR 'levosimendan'/exp/mj | 443,222 |
| #24 | 'catehcolamin*':ti,ab OR 'inotrop*':ti,ab OR 'adrenalin*':ti,ab OR 'epinephrin*':ti,ab OR 'noradrenalin*':ti,ab OR 'nor-adrenalin*':ti,ab OR 'norepinephrin*':ti,ab OR 'nor-epinephrin*':ti,ab OR 'dopamin*':ti,ab OR 'dobutamin*':ti,ab OR 'milrinon*':ti,ab OR 'simendan*':ti,ab OR 'levosimendan*':ti,ab) | 420,043 |
| #25 | #1 OR #2 | 95,199 |
| #26 | #3 OR #4 OR #5 OR #6 OR #7 OR #8 OR #9 OR #10 OR #11 OR #12 OR #13 OR #14 OR #15 OR #16 OR #17 OR #18 OR #19 OR #20 OR #21 OR #22 OR #23 OR #24 | 2,393,780 |
| #27 | #25 AND #26 | 4,108 |

### Appendix 6. Search strategy: Cochrane Library

Hits: 1167 (CDSR = 20, CENRAL = 1147)
Date performed: 27 March 2024

| Search line | Search | Results (n) |
| --- | --- | --- |
| #1 | MeSH descriptor: [Pulmonary Embolism] explode all trees | 1519 |
| #2 | (pulmonary OR lung OR venous) NEAR/2 (embol* OR thromb* OR clot) OR PE OR VTE; ti,ab,kw | 18,741 |
| #3 | #1 OR #2 | 18,742 |
| #4 | MeSH descriptor: [Diuretics] explode all trees | 4,107 |
| #5 | MeSH descriptor: [Vasodilator Agents] explode all trees 4619 | 4,619 |
| #6 | MeSH descriptor: [Nitric Oxide] explode all trees 2751 | 2,751 |
| #7 | MeSH descriptor: [Nitroglycerin] explode all trees | 2,275 |
| #8 | MeSH descriptor: [Nitroprusside] explode all trees | 682 |
| #9 | MeSH descriptor: [Prostaglandins] explode all trees | 7,509 |
| #10 | MeSH descriptor: [Phosphodiesterase Inhibitors] explode all trees | 1,651 |
| #11 | MeSH descriptor: [Endothelin Receptor Antagonists] explode all trees | 375 |
| #12 | MeSH descriptor: [Soluble Guanylyl Cyclase] explode all trees | 31 |
| #13 | MeSH descriptor: [Histamine Antagonists] explode all trees | 3,295 |
| #14 | MeSH descriptor: [Anti-Inflammatory Agents, Non-Steroidal] explode all trees | 9,653 |
| #15 | MeSH descriptor: [Vasoconstrictor Agents] explode all trees | 2,272 |
| #16 | MeSH descriptor: [Cardiotonic Agents] explode all trees | 1,464 |
| #17 | MeSH descriptor: [Catecholamines] explode all trees | 12,745 |
| #18 | MeSH descriptor: [Adrenergic Agonists] explode all trees | 4,025 |
| #19 | MeSH descriptor: [Milrinone] explode all trees | 238 |
| #20 | MeSH descriptor: [Simendan] explode all trees | 348 |
| #21 | (diure* OR furosemide OR frusemide OR vasodilat* OR hydralazine* OR hydrallazin* OR hydrazinophtalazine OR hydrazinophthalazine OR hydrazinophtalizine OR dralzine OR hydralacin OR hydralazine OR hypophthalin OR hypoftalin OR hydrazinophthalazine OR 1‐hydrazinophthalazine OR apressin OR nepresol OR apressoline OR apresoline OR apresolin OR alphapress OR alazine OR idralazina OR lopress OR plethorit OR praeparat OR nitric* OR inhaled-nitr* OR nitroprussid* OR nitro-prussid* OR sodium-nitr* OR nitrat* OR glyceryltrin* OR glyceryl-trin* OR tri-nitr* OR glyceryl-tri-nitr* OR nitroglycer* OR nitro-glycer* OR prostaglandin* OR epoprostenol OR prostacyclin* OR prostanoid* OR iloprost OR treprostinil OR selexipag OR phosphodiesterase*OR phospho-diest* OR phospho-di-est* OR sildenafil OR tadalafil OR dipyridamole OR persantin* OR (endothelin NEXT receptor*) OR endothelin-receptor* OR bosentan OR macitentan OR ambrisentan OR guanylyl* OR guanylat* OR riociguat* OR histamin* OR antihistamin* OR anti-histamin* OR chlorphen* OR meclofen* OR diclofena* OR ibuprofen* OR NSAID* OR (non-steroid* NEXT anti*) OR (nonsteroid* NEXT anti*) OR vasopressin* OR arginine-vasopress* OR terlipressin* OR phenylephrin* OR catecholamine* OR introp* OR adrenalin* OR epinephrin* OR noradrenalin* OR nor-adrenalin* OR norepinephrin* OR nor-epinephrin* OR dopamin* OR dobutamin* OR milrinon* OR simendan* OR levosimendan*):ti,ab,kw | 116,026 |
| #22 | (fluid* OR volume):ti | 15,099 |
| #23 | #4 OR #5 OR #6 OR #7 OR #8 OR #9 OR #10 OR #11 OR #12 OR #13 OR #14 OR #15 OR #16 OR #17 OR #18 OR #19 OR #20 OR #21 OR #22 | 144,595 |
| #24 | #3 AND #23 | 1167 |

### Appendix 7. Iterative search: Embase

Our search revealed further agents that have been investigated in the haemodynamic support of PE that were not adequately included the search strategy. We performed a further search to include these.

| Search line | Search | Results (n) |
| --- | --- | --- |
| #1 | ((('lung' OR 'pulmonary' OR 'venous') NEAR/2 ('embol*' OR 'thromb*' OR 'clot')):ti) OR 'pulmonary-embol*':ti OR 'pulmonary-thromb*':ti OR 'lung-thromb*':ti OR 'lung-embol*':ti OR 'venous-embol*':ti OR 'venous-thromb*':ti OR 'venothromb*':ti OR 'venoembol*':ti OR 'veno-thromb*':ti OR 'veno-embol*':ti OR 'pe':ti OR 'vte':ti OR 'lung embolism’/mj | 95,617 |
| #2 | 'chlorpromazine'/de OR 'doxycycline'/de OR 'tempol'/de OR 'arginine'/de OR 'isothiourea'/de OR 'aminoguanidine'/de OR 'diethylenetriamine nonoate'/de OR 'ketanserin'/de OR 'adrenomedullin'/de OR 'imidazole'/de OR 'diltiazem'/de | 279,068 |
| #3 | #1 AND #2 | 331 |

Flow diagram:

Search = 331

Title/abstract screen = 20 (264 irrelevant)

After removing those already discovered = 12 (8 already discovered)

After full text review = 9 (2: irretrievable, 1: wrong outcomes)

To include (additional to formal search) = 9

### Appendix 8. Validation of Embase search strategy

Our search strategy included an exhaustive use of MeSH/Emtree terms, but due to the very broad search strategy, some limitations were required. First, for the ‘key-word’ component of our search, we limited this to ‘title-only’ for the ‘PE concept’ component of our search strategy when searching the Medline, CINAHL and Emtree databases (but included title-abstract-keyword search for Cochrane Database). All other keywords (ie haemodynamic support agents) were searched for in titles and abstracts. Secondly, for the Emtree database where relevant, breadth was limited by not selecting the ‘[search] as broad as possible’ (ie ‘/br’) function in the Emtree interface. This was done to limit the otherwise very large number of irrelevant search results. We validated this strategy with absent breadth functions by screening for any eligible articles among a sample (the first 1000 articles sorted by ‘relevance’) of each [ex-limitation] NOT [main search] search as described below.

Aim:

To confirm that increasing the sensitivity of our search strategy through expanded search functions would not identify additional studies that met out eligibility criteria.

Purpose:

To reduce the number of irrelevant search results.

Methods:

Using the Embase search interface, we performed the following test searches; 1) expanded keyword search for pulmonary embolism to additionally search abstracts (appendix table 7.1), and 2) expanded Emtree term ‘lung embolism’ using the function “[search] as broad as possible /br” (appendix table 7.2). We then combined each of these search strategies using the Boolean operator NOT with our final search strategy (to exclude those already discoverable). We then screened titles and abstracts of the first 1000 results of each search (sorted by the Embase function ‘most to least relevant’).

Results:

The expanded keyword search to the abstract field identified an additional 13,319 results, and the using the Emtree function “as broad as possible */br*” identified an additional 4,502 results, that were not already discoverable by our search strategy. We found zero out of 1000 article sample (in order of ‘most relevant’ by Embase engine) met our eligibility criteria in either of these two test searches.

Conclusion:

Our final search strategy is likely sensitive enough to achieve its aim.

Appendix table 8.1: Expanded Embase search results to include abstract keyword search for pulmonary embolism not already discoverable by our strategy.

| Search line | Search | Results |
| --- | --- | --- |
| #1 | *Embase search as detailed in Appendix 5* | 4,108 |
| #2 | (((('lung' OR 'pulmonary' OR 'venous') NEAR/2 ('embol*' OR 'thromb*' OR 'clot')):ti,ab) OR 'pulmonary-embol*':ti,ab OR 'pulmonary-thromb*':ti,ab OR 'lung-thromb*':ti,ab OR 'lung-embol*':ti,ab OR 'venous-embol*':ti,ab OR 'venous-thromb*':ti,ab OR 'venothromb*':ti,ab OR 'venoembol*':ti,ab OR 'veno-thromb*':ti,ab OR 'veno-embol*':ti,ab OR 'pe':ti,ab OR 'vte':ti,ab) AND ('diuretic agent'/exp/mj OR 'diure*':ti,ab OR 'frusemide':ti,ab OR 'furosemide':ti,ab OR fluid:ti OR volume:ti OR 'hydralazine'/exp/mj OR 'vasodilat*':ti,ab OR hydralazin*:ti,ab OR hydrallazin*:ti,ab OR hydrazinophtalazine:ti,ab OR hydrazinophtalizine:ti,ab OR dralzine:ti,ab OR hydralacin:ti,ab OR hydralazine:ti,ab OR hypophthalin:ti,ab OR hypoftalin:ti,ab OR hydrazinophthalazine:ti,ab OR 1‐hydrazinophthalazine:ti,ab OR apressin:ti,ab OR nepresol:ti,ab OR apressoline:ti,ab OR apresoline:ti,ab OR apresolin:ti,ab OR alphapress:ti,ab OR alazine:ti,ab OR idralazina:ti,ab OR lopress:ti,ab OR plethorit:ti,ab OR praeparat:ti,ab OR 'nitric oxide'/exp/mj OR 'nitroprusside sodium'/exp/mj OR 'glyceryl trinitrate'/exp/mj OR nitric*:ti,ab OR nitroprussid*:ti,ab OR 'prussid*':ti,ab OR nitrat*:ti,ab OR nitroglycer*:ti,ab OR 'nitro-glycer*':ti,ab OR 'glyceryl*':ti,ab OR 'prostanoid'/exp/mj OR 'iloprost'/exp/mj OR 'prostacyclin'/exp/mj OR prostaglandin*:ti,ab OR epoprostenol:ti,ab OR prostacyclin*:ti,ab OR prostanoid*:ti,ab OR iloprost:ti,ab OR treprostinil:ti,ab OR selexipag:ti,ab OR 'phosphodiesterase inhibitor'/exp/mj OR phosphodiesterase*:ti,ab OR 'phospho diest*':ti,ab OR 'phospho di est*':ti,ab OR sildenafil:ti,ab OR tadalafil:ti,ab OR dipyridamole:ti,ab OR persantin*:ti,ab OR 'endothelin receptor antagonist'/exp/mj OR 'endothelin receptor*':ti,ab OR bosentan:ti,ab OR macitentan:ti,ab OR ambrisentan:ti,ab OR 'guanylate cyclase'/exp/mj OR guanylyl*:ti,ab OR guanylat*:ti,ab OR riociguat*:ti,ab OR 'antihistaminic agent'/exp/mj OR histamin*:ti,ab OR antihistamin*:ti,ab OR 'anti histamin*':ti,ab OR chlorphen*:ti,ab OR 'nonsteroid antiinflammatory agent'/exp/mj OR meclofen*:ti,ab OR diclofena*:ti,ab OR ibuprofen*:ti,ab OR nsaid*:ti,ab OR 'non-steroid* anti*':ti,ab OR 'nonsteroid* anti*':ti,ab OR 'vasopressin'/exp/mj OR 'terlipressin'/exp/mj OR 'phenylephrine'/exp/mj OR vasopressin*:ti,ab OR 'arginine vasopress*':ti,ab OR terlipressin*:ti,ab OR phenylephrin*:ti,ab OR 'cardiotonic agent'/exp/mj OR 'catecholamine'/exp/mj OR 'adrenergic receptor stimulating agent'/exp/mj OR 'dopamine receptor stimulating agent'/exp/mj OR 'milrinone'/exp/mj OR 'levosimendan'/exp/mj OR 'catehcolamin*':ti,ab OR 'inotrop*':ti,ab OR 'adrenalin*':ti,ab OR 'epinephrin*':ti,ab OR 'noradrenalin*':ti,ab OR 'nor-adrenalin*':ti,ab OR 'norepinephrin*':ti,ab OR 'nor-epinephrin*':ti,ab OR 'dopamin*':ti,ab OR 'dobutamin*':ti,ab OR 'milrinon*':ti,ab OR 'simendan*':ti,ab OR 'levosimendan*':ti,ab) | 16,973 |
| #3 | #2 NOT #1 | 13,319 |

Appendix table 8.2: Expanded Embase search results to include Embase function “[search] as broad as possible /br” for pulmonary embolism not already discoverable by our strategy.

| Search line | Search | Results |
| --- | --- | --- |
| #1 | *Embase search as detailed in Appendix 5* | 4,108 |
| #2 | (((('lung' OR 'pulmonary' OR 'venous') NEAR/2 ('embol*' OR 'thromb*' OR 'clot')):ti) OR 'pulmonary-embol*':ti OR 'pulmonary-thromb*':ti OR 'lung-thromb*':ti OR 'lung-embol*':ti OR 'venous-embol*':ti OR 'venous-thromb*':ti OR 'venothromb*':ti OR 'venoembol*':ti OR 'veno-thromb*':ti OR 'veno-embol*':ti OR 'pe':ti OR 'vte':ti OR **‘lung embolism’/br**) AND ('diuretic agent'/exp/mj OR 'diure*':ti,ab OR 'frusemide':ti OR 'furosemide':ti OR fluid:ti OR volume:ti OR 'hydralazine'/mj/exp OR 'vasodilat*':ti,ab OR hydralazin*:ti,ab OR hydrallazin*:ti,ab OR hydrazinophtalazine:ti,ab OR hydrazinophtalizine:ti,ab OR dralzine:ti,ab OR hydralacin:ti,ab OR hydralazine:ti,ab OR hypophthalin:ti,ab OR hypoftalin:ti,ab OR hydrazinophthalazine:ti,ab OR 1‐hydrazinophthalazine:ti,ab OR apressin:ti,ab OR nepresol:ti,ab OR apressoline:ti,ab OR apresoline:ti,ab OR apresolin:ti,ab OR alphapress:ti,ab OR alazine:ti,ab OR idralazina:ti,ab OR lopress:ti,ab OR plethorit:ti,ab OR praeparat:ti,ab OR 'nitric oxide'/exp/mj OR 'nitroprusside sodium'/exp/mj OR 'glyceryl trinitrate'/exp/mj OR nitric*:ti,ab OR nitroprussid*:ti,ab OR 'prussid*':ti,ab OR nitrat*:ti,ab OR nitroglycer*:ti,ab OR 'nitro-glycer*':ti,ab OR 'glyceryl*':ti,ab OR 'prostanoid'/exp/mj OR 'iloprost'/exp/mj OR 'prostacyclin'/exp/mj OR prostaglandin*:ti,ab OR epoprostenol:ti,ab OR prostacyclin*:ti,ab OR prostanoid*:ti,ab OR iloprost:ti,ab OR treprostinil:ti,ab OR selexipag:ti,ab OR 'phosphodiesterase inhibitor'/exp/mj OR phosphodiesterase*:ti,ab OR 'phospho diest*':ti,ab OR 'phospho di est*':ti,ab OR sildenafil:ti,ab OR tadalafil:ti,ab OR dipyridamole:ti,ab OR persantin*:ti,ab OR 'endothelin receptor antagonist'/exp/mj OR 'endothelin receptor*':ti,ab OR bosentan:ti,ab OR macitentan:ti,ab OR ambrisentan:ti,ab OR 'guanylate cyclase'/exp/mj OR guanylyl*:ti,ab OR guanylat*:ti,ab OR riociguat*:ti,ab OR 'antihistaminic agent'/exp/mj OR histamin*:ti,ab OR antihistamin*:ti,ab OR 'anti histamin*':ti,ab OR chlorphen*:ti,ab OR 'nonsteroid antiinflammatory agent'/exp/mj OR meclofen*:ti,ab OR diclofena*:ti,ab OR ibuprofen*:ti,ab OR nsaid*:ti,ab OR 'non-steroid* anti*':ti,ab OR 'nonsteroid* anti*':ti,ab OR 'vasopressin'/exp/mj OR 'terlipressin'/exp/mj OR 'phenylephrine'/exp/mj OR vasopressin*:ti,ab OR 'arginine vasopress*':ti,ab OR terlipressin*:ti,ab OR phenylephrin*:ti,ab OR 'cardiotonic agent'/exp/mj OR 'catecholamine'/exp/mj OR 'adrenergic receptor stimulating agent'/exp/mj OR 'dopamine receptor stimulating agent'/exp/mj OR 'milrinone'/exp/mj OR 'levosimendan'/exp/mj OR 'catehcolamin*':ti,ab OR 'inotrop*':ti,ab OR 'adrenalin*':ti,ab OR 'epinephrin*':ti,ab OR 'noradrenalin*':ti,ab OR 'nor-adrenalin*':ti,ab OR 'norepinephrin*':ti,ab OR 'nor-epinephrin*':ti,ab OR 'dopamin*':ti,ab OR 'dobutamin*':ti,ab OR 'milrinon*':ti,ab OR 'simendan*':ti,ab OR 'levosimendan*':ti,ab) | 8,610 |
| #3 | #2 NOT #1 | 4,502 |
